# Association of high LDL concentrations with erectile dysfunction from a Mendelian randomization study

**DOI:** 10.1038/s41598-023-49771-1

**Published:** 2023-12-14

**Authors:** Quan Zhu, Yao Tan, Xuyan Zou, Liqing Lu

**Affiliations:** 1 Xiangya Hospital, Central South University, Changsha, 410008 Hunan China; 2 Department of Ophthalmology, The Third Xiangya Hospital, Central South University, No. 138 Tongzipo Road, Yuelu District, Changsha, 410013 Hunan China; 3 The First Affiliated Hospital of Jinan University, Guangzhou, 510630 China

**Keywords:** Genetic association study, Urogenital diseases

## Abstract

Lipid metabolism plays a key role in erectile dysfunction. Our purpose was to evaluate the influence of lipid-lowering drugs on erectile dysfunction employing a two-sample Mendelian randomization (MR) study. Genetic instruments were employed to represent the exposure of lipid-lowering drugs. Inverse variance-weighted MR (IVWMR) was employed to calculate the estimation of effects. IVW-MR analysis showed that the positive relationship between the expression of HMGCR and the risk of erectile dysfunction (odds ratio [OR] = 1.27, 95% confidence interval [CI] 1.03–1.57; *p* = 0.028). No significant relationship was detected between NPC1L1, PSK9 expression and erectile dysfunction. This MR study suggested that HMGCR inhibitors are a more desirable treatment modality for patients with ED.

## Introduction

Erectile dysfunction (ED), defined as the inability to develop or maintain a penile erection during sexual activity, is a common disorder in men. The prevalence increases with age, with a 70% prevalence at age 70 years^1^. Research has reported an overall ED prevalence of 49.69% in mainland China^2^, and an increasing number of men under the age of 40 are suffering from ED^3^. ED can be divided into three categories based on etiology: psychological, organic, and mixed. More than half of ED patients have organic changes^4^. Because the penis has a special vascular network, vascular factors, especially the status of the penile arteries, are more important in organic ED^5^. Atherosclerosis, endothelial dysfunction and inflammation, which are pathological factors affecting vascular function, can lead to atherogenic ED^6^. Therefore ED is also considered as a manifestation of systemic atherosclerosis^7^.

Dyslipidemia is an important risk factor for endothelial dysfunction^8^. The low-density lipoprotein (LDL) is considered one of the strongest predictors of atherosclerosis and endothelial dysfunction, and lowering LDL is essential for the prevention and treatment of atherosclerosis^9^. LDL mediates atherosclerosis by entering the vessel wall through endothelial cells, leading to oxidation of LDL by endothelial cells, which in turn leads to endothelial dysfunction^10^. LDL elevation can cause endothelial dysfunction in the penile arteries of mice and further induce the development of ED^11^. Due to the small average diameter of penile arteries, a decrease in blood flow is more likely to occur in the presence of plaque. Moreover, erection of the penis requires a lot of dilation and dysfunction of the vascular endothelium in the cavernous vascular bed has a great impact on the cavernous arteries^6^. Thus, lipid abnormalities are an important cause of ED.

ED is often accompanied by cardiovascular disease, and dyslipidemia is an important risk factor for cardiovascular disease. Therefore, the use of lipid-lowering drugs has gradually increased in ED patients^12^. 3-Hydroxy-3-methylglutaryl coenzyme A reductase (HMG-CoA reductase (HMGCR)) is the rate-limiting enzyme in the valproic acid pathway and is responsible for the synthesis of cholesterol and steroid hormones, and HMG-CoA inhibitors (statins) are commonly used clinically as lipid-lowering drugs to reduce circulating total cholesterol and low-density lipoprotein cholesterol (LDL-C) levels^13–15^. Clinical two other drugs approved for common use that target cholesterol metabolism include ezetimibe (targeting Niemann-pickc1-like protein (NPC1L1)) and proprotein convertase kwashiorkor/kexin type 9 (PCSK9) inhibitors. They enhance LDL-C uptake by reducing intestinal absorption of cholesterol or by increasing cell membrane expression of the ldl receptor (LDLR)^16^. However, the effect of lipid-lowering drugs on erectile function in patients with or without ED is not known.

Mendelian randomization (MR) analysis utilizes the inherent properties of common genetic variants to provide a causal validation method that is not susceptible to social, environmental, and behavioral factors^17^, and has become a common way to explore potential causal relationships between exposure factors and disease^18^. This article explores the relationship between ED's and three different targets of lipid-lowering drugs using two sample pools: 3-hydroxy-3 methylglutaryl-CoA reductase (HMGCR) inhibitors, Niemann-Pick C1-like 1 (NPC1L1) inhibitors, and PCSK9 inhibitors.

## Materials and method

### Research methodology

This two-sample MR analysis is predicated upon the utilization of open-access, anonymized, aggregate data extracted from previously conducted Genome-Wide Association Studies (GWASs) (Table 1). All foundational investigations acquired ethical clearance from their respective Institutional Review Boards, with relevant references duly noted, and participating individuals provided informed consent.

**Table 1:** Information of all GWAS summary data.

Traits	Resource	Sample size	Population ancestry	Data download
LDL cholesterol	Global lipids genetics consortium	173,082	European	https://csg.sph.umich.edu/abecasis/public/lipids2013/jointGwasMc_LDL.txt.gz
Erectile dysfunction	The IEU GWAS database	Number of cases: 6175 Number of controls: 217,630	European	https://gwas.mrcieu.ac.uk/datasets/ebi-a-GCST006956/
Coronary heart disease	CARDIoGRAMplusC4D consortium	Number of cases:60,801 Number of controls: 123,504	European	http://www.cardiogramplusc4d.org/media/cardiogramplusc4d-consortium/data-downloads/mi.additive.Oct2015.pub.zip

In our study, we diligently followed three key assumptions when selecting instrumental variables (IVs) for MR analysis: (1) The SNP (instrument) is correlated with the exposure (lipid-lowering drugs). (2) The SNP is independent of any confounders between the exposure and outcome. 3) The SNP influences the outcome (ED) solely via its relationship with the exposure.

### Genetic instrument selection

This investigation encompassed three classifications of FDA-endorsed lipid-lowering pharmaceuticals as exposure factors: HMGCR inhibitors, PCSK9 inhibitors, and the NPC1L1 inhibitor.

**Figure 1:**
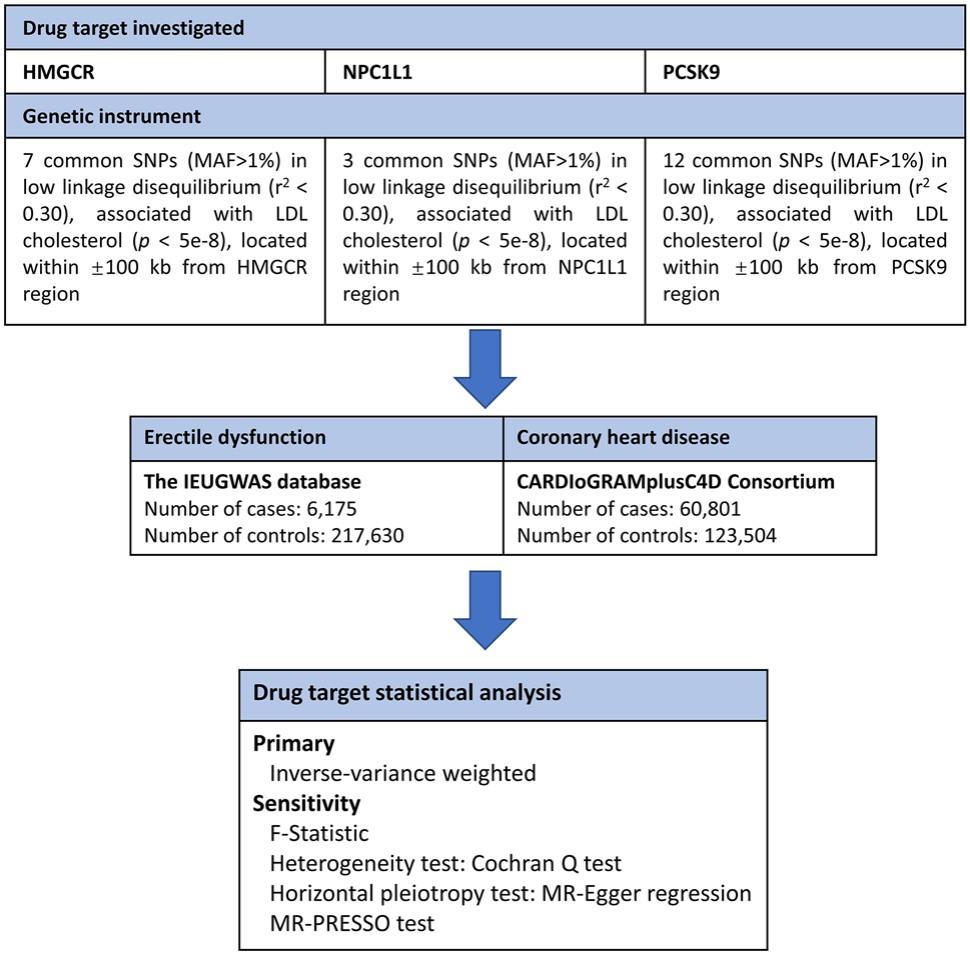
Flowsheet of Mendelian randomisation in this study.

To corroborate the associations discerned through the employment of eQTLs as genetic instruments, we adopted a strategy that involved selecting SNPs situated within 100 kb windows surrounding each drug's target gene. These SNPs were associated with LDL cholesterol concentrations at a genome-wide significance threshold (p < 5.0 × 10^−8^) and served as proxies for exposure to lipid-lowering medications. The GWAS summary data related to LDL cholesterol levels, procured from the Global Lipids Genetics Consortium (GLGC) and comprising a sample size of 173,082 participants, was employed to pinpoint these SNPs^19^ (Supplementary Table S1). The screening flowsheet is shown in Fig. 1.

The selection of these genetic instruments was based on their association with LDL cholesterol concentrations at a genome-wide significance threshold (p < 5.0 × 10^–8^). This criterion ensures that the selected SNPs are robustly associated with the exposure. While each individual SNP might explain a small fraction of the variance in LDL concentration, the combined set of SNPs, especially considering their strong statistical association with LDL cholesterol (evidenced by their p-values), ensures a reliable genetic instrument.

For HMGCR inhibitors, seven prevalent SNPs (MAF > 1%) were pinpointed within ± 100 kb windows encompassing the HMGCR region, exhibiting low linkage disequilibrium (r^2^ < 0.30) and a significant association (p < 5.0 × 10^−8^). Similarly, PCSK9 inhibitors had twelve common SNPs, and NPC1L1 inhibitors had three common SNPs, meeting the same criteria within ± 100 kb windows of their respective regions.

The statistical evaluations comprised a primary analysis utilizing inverse-variance-weighted MR (IVW-MR), in addition to sensitivity analyses encompassing the F-Statistic, positive control assessment, linkage disequilibrium assessment, horizontal pleiotropy examination, heterogeneity testing, and MR-Egger regression.

### Sources of outcome data

GWAS summary-level data concerning erectile dysfunction outcomes, sourced from the IEU GWAS database, which includes a sample size of 223,805, with 6175 cases and the remaining being controls^20^.

### Statistical analysis

#### Primary MR analysis

When utilizing genetic variants associated with LDL cholesterol concentrations as instruments, the IVW-MR method was implemented to amalgamate effect estimates. Allele harmonization and subsequent analysis were conducted using version 4.2.3 of the TwoSampleMR package within the R software environment.

#### Sensitivity analysis

We evaluated the strength of the SNPs used as instruments by calculating the F-statistic, including only SNPs with an F-statistic greater than 10 in order to mitigate the potential for weak instrument bias^21^. To validate both genetic instruments, we performed positive control analyses. Given that lipid-lowering drugs are known to effectively reduce LDL cholesterol levels, we investigated the relationship between the exposures of interest and LDL cholesterol levels as a positive control study for the eQTLs-based instruments. In the case of the instrument derived from the LDL cholesterol GWAS, we carried out a positive control study by examining the association between the exposures of interest and coronary heart disease. This was done because coronary heart disease is the primary indication for lipid-lowering medications.

In the IVW-MR method, we evaluated heterogeneity using the Cochran Q test, wherein a p-value below 0.05 indicates the presence of heterogeneity^22^. To assess the potential for horizontal pleiotropy among the SNPs utilized as instrumental variables, we utilized MR-Egger regression and Mendelian Randomization Pleiotropy RESidual Sum and Outlier (MR-PRESSO) analysis. In MR-Egger regression, the intercept term serves as a valuable indicator for assessing directional horizontal pleiotropy, whereby a p-value below 0.05 suggests the presence of horizontal pleiotropy^23^. MR-PRESSO analysis can identify horizontal pleiotropic outliers and offer adjusted estimates, with a p-value below 0.05 for the Global test indicating the presence of horizontal pleiotropic outliers^24^. All of the aforementioned analyses were conducted utilizing R software, version 4.2.3.

## Results

### SNP selection and validation

Using the ED data from the IEU GWAS database, we selected specific SNPs to proxy LDL lowering through the inhibition of HMGCR, NPC1L1, and PCSK9. Specifically: For HMGCR, we identified 7 SNPs to proxy HMG-CoA reductase inhibition: rs10066707, rs10515198, rs12659791, rs12916, rs3804231, rs3857388, rs72633962. For NPC1L1, 3 SNPs were selected to proxy its inhibition: rs2073547, rs217386, and rs7791240. For PCSK9, 12 SNPs were chosen to proxy its inhibition: rs10493176, rs11206510, rs11206514, rs11583974, rs11591147, rs12067569, rs2479394, rs2479409, rs2495495, rs4927193, rs572512, and rs585131 (Supplementary Table S1). Moreover, Supplementary Table S2 results showed that a significant relationship between genetically proxied drug targets and CAD, which were regarded as the positive control analyses, assuring the efficacy of the genetic instruments.

### Analysis using the two-sample MR

**Figure 2:**
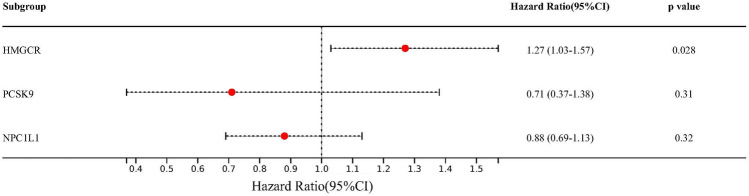
Inverse-variance-weighted Mendelian randomization (IVW-MR) association between the gene HMGCR, PCSK9, or NPC1L1 and erectile dysfunction.

IVW-MR analysis results showed that the positive relationship between the expression of HMGCR and the risk of erectile dysfunction (odds ratio [OR] = 1.27, 95% confidence interval [CI] 1.03–1.57; *p* = 0.028), suggesting that lower HMGCR expression reduced the risk of erectile dysfunction (Fig. 2 and Table 2). No significant relationship was detected between NPC1L1, PSK9 expression and erectile dysfunction.

**Table 2:** Associations between genetically proxied inhibition of HMGCR, NPC1L1, or PCSK9 and erectile dysfunction.

Method	nSNPs	Beta	SE	OR (95%CI)	*P* value
HMGCR					
Inverse variance weighted	7	0.24	0.11	1.27 (1.03–1.57)	2.8e−02
MR Egger	7	0.18	0.84	1.20 (0.23–6.25)	0.84
Weighted median	7	0.18	0.21	1.19 (0.79–1.80)	0.40
NPC1L1					
Inverse variance weighted	3	− 0.34	0.34	0.71 (0.37–1.38)	0.31
MR Egger	3	3.27	2.53	26.32 (0.19–3742.92)	0.42
Weighted median	3	− 0.13	0.39	0.87 (0.41–1.87)	0.73
PCSK9					
Inverse variance weighted	12	− 0.12	0.12	0.88 (0.69–1.13)	0.32
MR Egger	12	− 0.25	0.20	0.78 (0.52–1.16)	0.24
Weighted median	12	− 0.17	0.14	0.84 (0.64–1.11)	0.22

### Sensitivity analysis

**Figure 3:**
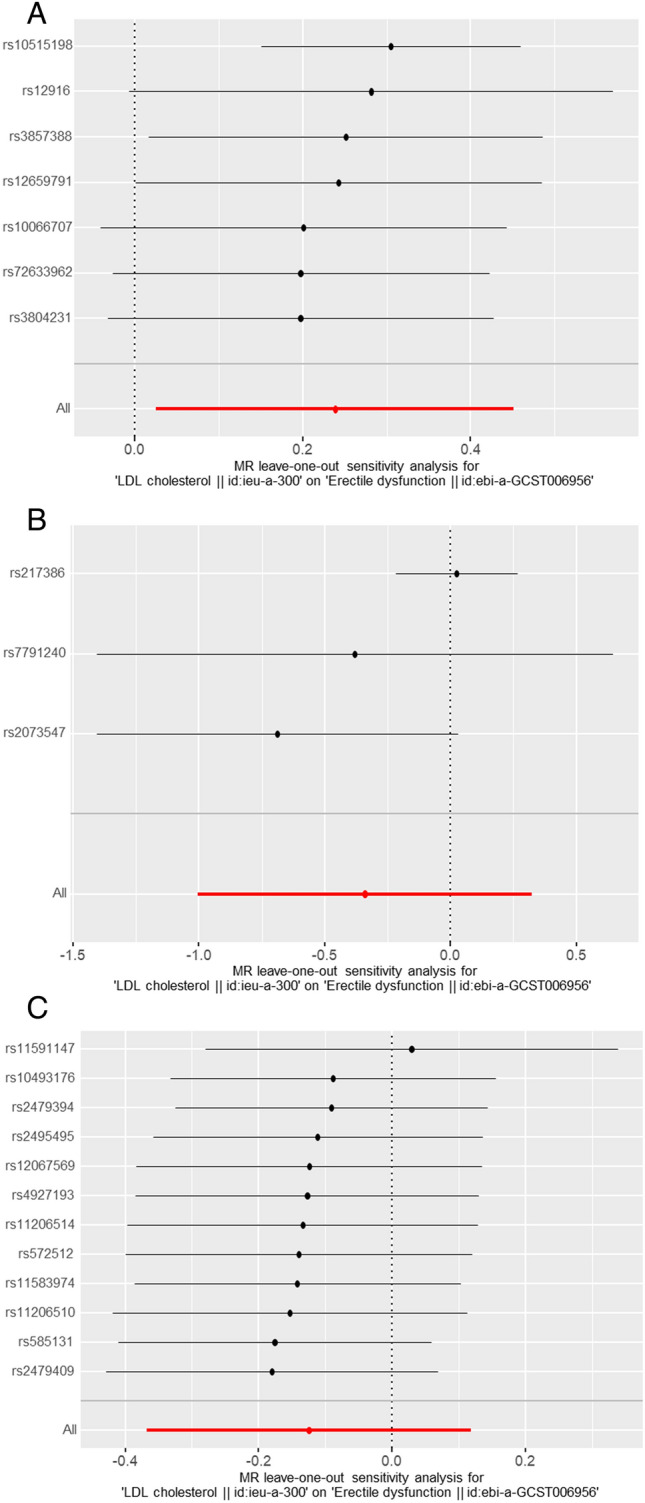
Forest plot of the LDL-related gene SNP and pooled MR estimates the erectile dysfunction risk. (**A**) HMGCR; (**B**) PCSK9; (**C**) NPC1L1.

A lack of causal association remained in all sensitivity analyses (all p > 0.05). Likewise, there was no clear evidence of heterogeneity (all p > 0.05) or pleiotropy (all p values for intercept > 0.05) regarding HMGCR, NPC1L1 and PCSK9 in erectile dysfunction and positive control coronary heart disease (Table 3). Moreover, the leave-one-out analysis plot results certified that the above results were unchanged by the removing of any SNP and were quite robust (Fig. 3).

**Table 3:** Sensitive analyses for genetically proxied inhibition of HMGCR, NPC1L1, or PCSK9.

Outcome	*P* value for MR-Egger intercept	MR Egger* P*-value for Cochran Q test	Inverse variance weighted* P*-value for Cochran Q test	*P* value for MR-PRESSO global test
HMGCR				
Erectile dysfunction	0.949	0.754	0.851	0.879
Coronary heart disease	0.272	0.675	0.585	0.574
NPC1L1				
Erectile dysfunction	0.386	0.841	0.348	NA
Coronary heart disease	0.728	0.967	0.901	NA
PCSK9				
Erectile dysfunction	0.445	0.130	0.141	0.164
Coronary heart disease	0.757	0.079	0.109	0.134

## Discussion

In the present study, increased HMGCR gene expression was associated with an increased risk of erectile dysfunction, suggesting that HMGCR inhibitors may reduce the risk of erectile dysfunction. The expression of NPC1L1 and PCSK9 was not significantly associated with ED.

Statins are the most commonly used HMGCR inhibitors in clinical practice and play an important role in lowering LDL and cholesterol and slowing the progression of atherosclerosis^25^. Increased production of LDL receptors is thought to be the main mechanism driving the action of statins^26^. Statins also have an ameliorating effect on platelet and vascular endothelial function^27^.However, statins still have some side effects, including statin-related muscle symptoms, new-onset type 2 diabetes, neurological and neurocognitive effects^28^.

Statins may improve penile erectile function in several ways; a reduction in LDL may improve endothelial function, and good endothelial function is important for penile erection. Moreover, statins may increase the availability of nitric oxide, which plays a key regulatory role in penile erection and resistance to oxidative stress. However, statins may also impair penile erectile function because they block reductase in the early stages of cholesterol biosynthesis, thereby reducing testosterone formation^29^. Testosterone is closely associated with sexual behavior, and it enhances the overall male sexual response^30^. In patients with reduced testosterone levels, their sexual desire and number of morning erections are significantly reduced^31^.

However, the effect of statins on ED in men remains controversial in current clinical studies. In 1812 men treated with statins, no association between statin use and the development of ED was found by testing testosterone and luteinizing hormone^32^. In contrast, in a study by BRUCKERT et al. patients who were treated with statins were more likely to suffer from ED^33^. And in another individual study, it was also found that some patients experienced decreased libido and significantly lower testosterone after taking different types of statins^34^. These opposite findings may be due to various differences in the age of initiation of statin therapy, the dose used, and the type of their underlying disease.

As for NPC1L1 and PCSK9 inhibitors, there was no significant correlation between them and ED in this study. We consider that on the one hand, it is because the lipid-lowering effect of NPC1L1 inhibitors is not as strong as that of statins; and PCSK9 inhibitors, although they have a strong lipid-lowering effect, have a low bioavailability and are expensive, which leads to the fact that both of them are usually used only by patients with poor lipids^35,36^. This problem of poor lipids may make the protective effect of NPC1L1 and PCSK9 inhibitors on ED not significant. On the other hand, the inhibition of the cholesterol biosynthesis process by statins may inhibit multiple actions of downstream products together, which may also be a factor in the ability of statins to exert ED protection, but this needs to be verified by more subsequent studies.

## Strengths and limitations

(1) Participants of European ancestry only were enrolled in the study; future studies of populations of other ethnicities are needed to test the generalizability of the current study's findings. (2) We utilized MR to reduce confounding in studying ED's association with lipid-lowering drugs. While stringent SNP selection and sensitivity analyses bolster our study, it's essential to acknowledge that potential biases, like horizontal pleiotropy, can't be fully eliminated. (3) Concerns arose over potential overlaps between IEU GWAS and our eQTL-derived GWASs. Despite verifying distinct cohorts, minor overlaps are possible. This might introduce biases, but our MR-Egger and MR-PRESSO analyses aim to control such biases. (4) Our MR analysis carefully chooses SNPs based on proximity to drug target genes and LDL cholesterol significance. While methods like the Cochran Q test help, the intrinsic variability remains. Despite robust techniques like IVW-MR and MR-Egger regression, potential biases from SNP variability persist. While our results offer valuable insights, they warrant cautious interpretation, suggesting future research could benefit from expanded SNP criteria for enhanced robustness.

## Conclusion

In the present study, we reduced the risk of confounding by a Mendelian randomization study and found that HMGCR gene expression was positively associated with ED occurrence, whereas NPC1L1 and PCSK9 gene expression did not correlate with ED occurrence. It is suggested that the use of HMGCR inhibitors may reduce the occurrence of ED and that HMGCR inhibitors are a more desirable treatment modality for patients with cardiovascular disease combined with ED.

### Supplementary Information

Supplementary Tables.

## Data Availability

The original contributions presented in the study are included in the article/Supplementary Material, further inquiries can be directed to the corresponding authors.
